# Assessment of Dental Implants with Modified Calcium-Phosphate Surface in a Multicenter, Prospective, Non-Interventional Study: Results up to 50 Months of Follow-Up

**DOI:** 10.3390/jfb10010005

**Published:** 2019-01-11

**Authors:** Carles Subirà-Pifarré, Cristina Masuet-Aumatell, Carlos Rodado Alonso, Ricardo Medina Madrid, Cosimo Galletti

**Affiliations:** 1 Biomedical Research Institute (IDIBELL), L’Hospitalet de Llobregat, 08907 Catalonia, Spain; cmasuet@bellvitgehospital.cat; 2 Comprehensive Dentistry Department, Faculty of Dentistry, Universitat de Barcelona, L’Hospitalet de Llobregat (Barcelona), 08907 Catalonia, Spain; cosimo88a@gmail.com; 3 Preventive Medicine Department, University Hospital of Bellvitge, IDIBELL, L’Hospitalet de Llobregat (Barcelona), 08907 Catalonia, Spain; 4 Cimax Dr Carlos Rodado, 17002 Girona, Spain; drcarlosrodado@cimaxclinic.com; 5 Private Dental Clinic, 28821 Madrid, Spain; r.medina@clinica-famed.com

**Keywords:** dental implants, modified calcium-phosphate surface, observational study, routine clinical practice, survival rate, method of choice, osseointegrated implants

## Abstract

Prescription of implant treatments is very widespread at present, mainly due to the low rate of annual loss and, to date, few studies have assessed their survival in the routine clinical practice of dentistry. The purpose of this observational study was to evaluate the effectiveness of dental implants with a calcium-phosphate surface in the daily practice of dental clinics. A multicenter, prospective, non-interventional, observational study was performed, in which three experienced practitioners (one maxillofacial and two oral surgeons) inserted implants using standard external and internal hexagon connections in adult patients requiring ≥1 osseointegrated implants to replace missing teeth. Follow-up was performed for 24 months after implant loading. Two hundred and twelve subjects were included (51.5% men), with a mean age of 51.2 ± 11.90 years, in whom 544 implants were inserted. 87.2% of the patients received 1–4 implants. The preferred connection system was internal hexagon (73.5%). There were nine failures, with an interval survival rate (ISR) at 24 months of 100% and a cumulative survival rate (CSR) of 98.3%. In conclusion, implants with a modified calcium-phosphate surface are associated with a high rate of survival and may be considered a method of choice in clinical practice.

## 1. Introduction

The main reason for which treatment with endosseous dental implants is so widespread at present is the low annual implant loss rate, as has been demonstrated by recent controlled trials, systematic reviews and meta-analyses [1,2,3,4,5,6,7]. Nevertheless, implant treatment is not exempt from complications and failures, both at short- (1.4 to 2.7%) and long-term (1.6 to 2.72% after more than nine years) [2,8,9]. According to the literature, these complications and failures are higher among smokers and patients with an initial diagnosis of periodontitis [10,11,12,13,14,15,16].

To date, however, few reports have been published of studies in real-life conditions, in the everyday clinical practice of dentistry [17,18,19,20,21].

Generally speaking, it is known that the outcome of implant treatment basically depends on the design of the implant, the position in which it is placed, the surgical technique, the conditions of the patients and the type of prosthesis [22]. It has been estimated that at present dentists can choose from more than 1300 different types of implants, made from different materials, in different shapes, sizes, lengths and surface properties [23,24,25]. The Biomimetic Ocean implants (Avinent Implant System, Barcelona, Spain), with a modified calcium-phosphate surface, have an innovative geometry that adapts to the biological architecture of the bone promoting primary stability, preservation of the bone and esthetics, and it has positive angle platform switching to facilitate soft tissue adaptation. The implant is characterized by their biomimetic advanced surface, which is classified as moderately rough, and has a surface topography that features macro-roughness, achieved through a physical process, and microporosity obtained through a chemical process whereby calcium and phosphorus are incorporated into the titanium oxide thus creating an osteoconductive surface that enhances osseointegration [26].

To examine the survival of implants with a calcium-phosphate surface (Biomimetic Ocean implants), including short (7 and 8.5 mm) and long implants (≥10 mm), a prospective, non-interventional, observational study, with a 24-month follow-up, was designed in the routine clinical practice of dental clinics. The results showed that implants with a modified calcium-phosphate surface were clinically osseointegrated, which would be consistent with the high survival rates observed at 12-months and 24-months.

## 2. Results

A total of 212 subjects (51.5% men and 48.5% women) with 544 implants and a mean age of 51.2 ± 11.90 years were included. Demographic data, number of implants and oral health features of the patients are shown in Table 1.

**Figure 1:**
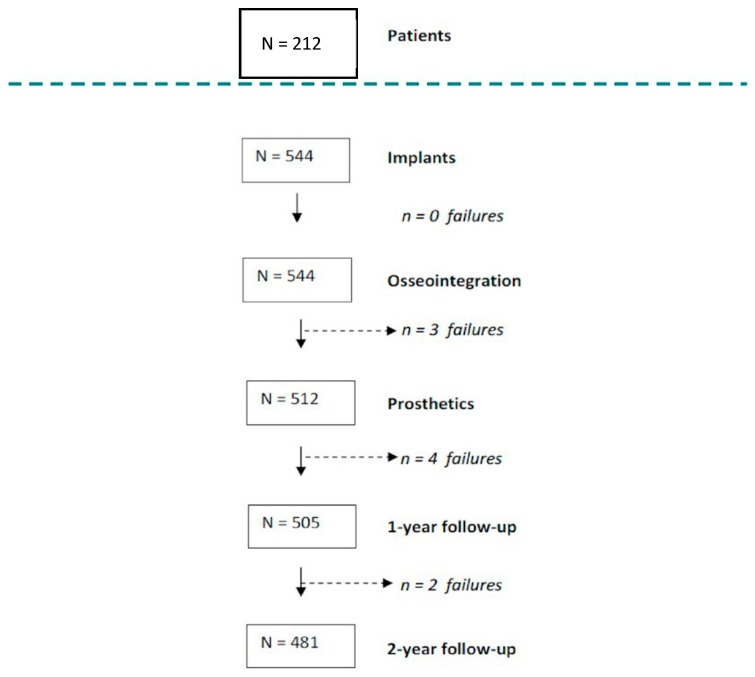
Flow-chart and follow-up of the implants (n = 544) study in 212 patients during 24 months. Number of failures that occurred at different stages of the study. Nine failures were recorded at different stages of the study: five during the osseointegration period and four after prosthetic loading.

As shown in Figure 1, nine failures were recorded at different stages of the study: five during the osseointegration period and four after prosthetic loading. A total of 11.6% of the implants could not be evaluated because of different reasons (dropouts). The main reasons for which patients did not continue the study were death, withdrawal from treatment due to economic reasons, or change of residence.

The main oral risk factors for failure were smoking (18.8%), controlled periodontal treatment (30.2%) and bruxism (35.8%), without statistically significant differences between patients with or without failures.

**Figure 2:**
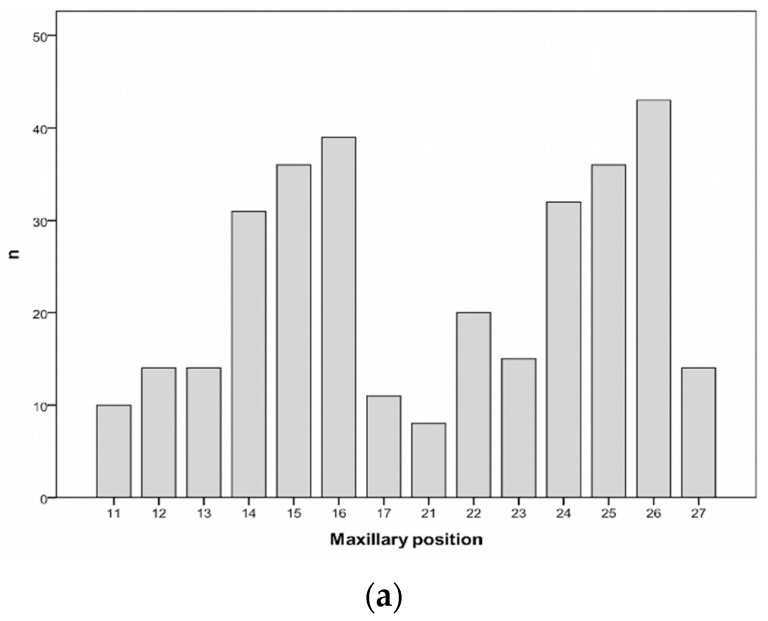

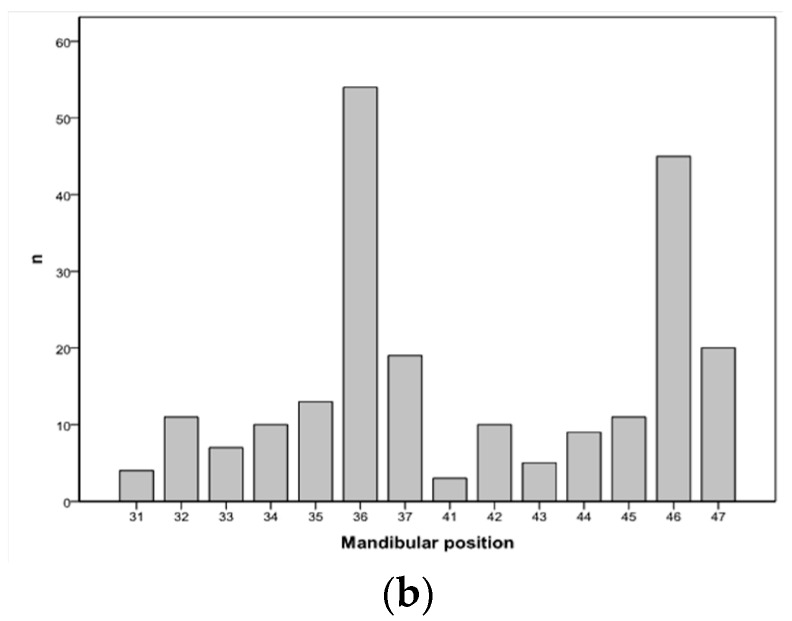
Distribution of the implants according to positioning. The number of implants per tooth position is depicted in the maxilla (**a**) and in the mandible (**b**), according to the World Dental Federation notation.

The majority of patients received between one and four implants (87.2%), while only three patients received 12 implants (Table 1). According to the distribution of the positioning, most of the implants were placed in the posterior region (Figure 2); 323 implants were placed in the maxilla (59.4%) and 221 in the mandible (40.6%). The most common placement site was 36 (9.9%) (lower left first molar), followed by 46 (8.3%) (lower right first molar).

Internal connections were chosen most frequently (73.5% of cases). The preferred diameter was 4.0 or 4.5 mm (68.7%) and the most frequently used length was 11.5 mm (42.8%) (Table 2).

Most of the implants (79%) were placed in bone density type II or III according to the Lekholm and Zarb classification system (Table 2). In the majority of cases, the bone had healed (69.5%) and in 23.9% of cases, it was regenerated (including bone regeneration prior to surgery: sinus lifts, alveolar ridge preservation, etc., as well as regeneration during the surgery itself).

Two-stage submerged healing was the technique selected in the majority of cases (83.6%), with time for osseointegration of more than three months (88.1%). Only 5.9% of the implants were loaded immediately.

In 93.8% (n = 510) of cases the implant platform was placed at the crestal level, as indicated by the implant protocol that was used; 5.3% were left in the subcrestal position (n = 29) and only 0.9% in the supracrestal position (n = 5).

The type of prostheses that were used most frequently were bridges (54.2%) together with individual crowns (29%), in both cases screwed directly to the implant, followed by cement-retained restorations (6%) and attachment-retained overdentures (4.6%) (Table 3). In 58.8% of cases, the design of the prosthesis was made using digital data acquisition and the material most often used for manufacturing was milled CoCr (52.8%).

**Figure 3:**
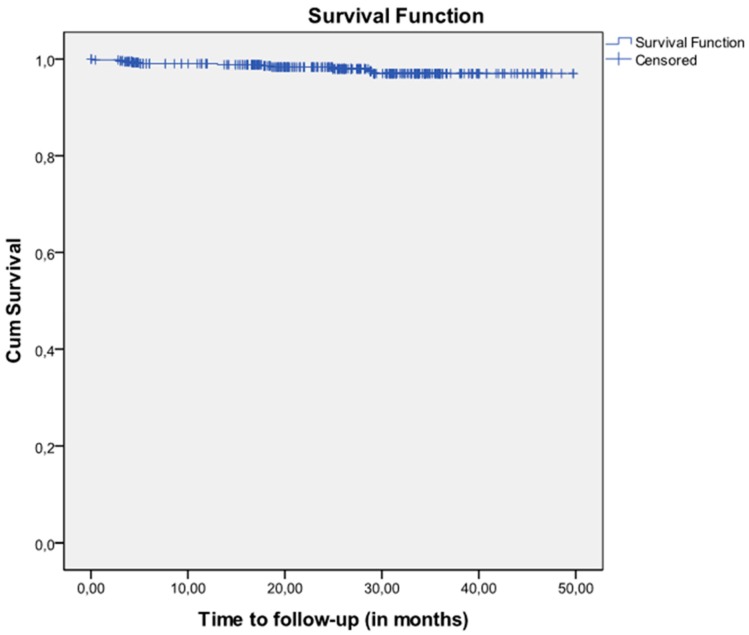
Implant survival Kaplan-Meier curves from start to end of follow-up (n = 544) to evaluate the effectiveness of the implant and its survival at two years (some implants were followed up to 50 months).

During the 24 months of follow-up nine failures were documented, which represents a rate of 98.32% cumulative survival over time, as shown in the Kaplan Meier survival curve in Figure 3.

Five implants failed during the period of osseointegration and four failed in the period after prosthetic loading, as shown in Table 4. Three were lost because of postoperative infection, another three due to fibro-osseous integration detected when the impression was made for the final prosthesis, one as a result of peri-implantitis (inflammation and destruction of soft and hard tissues surrounding dental implants) [27] and two were due to asymptomatic spontaneous avulsion of the implant (due to fibro-integration).

It can be seen that the CSR of 462 implants at 12 months was 99.04% (95% confidence interval -CI-: 98.16–99.93) and at 24 months the CSR for 334 implants was 98.32% (95% CI: 96.94–99.70) (Table 5).

It was also found that no patient or technique-related risk factors affected the survival results, demonstrating the predictability of the Avinent^®^ system in any real everyday clinical situation (Table 6). For this reason, a multivariate Cox regression analysis was not performed, since none of these factors or variables affected implant survival (*p* > 0.05).

## 3. Discussion

Although it may provide a standard design, materials and surface, any new system of dental implants must undergo a scientific assessment to validate proper performance in conventional clinical conditions.

The main objective of this multicenter, prospective, non-interventional, observational study was to describe the 24-month survival rate of implants with a modified calcium-phosphate surface, including short (7 and 8.5 mm) and long implants (≥10 mm), in daily clinical practice. The cohort that was followed represents a real sample of patients who were treated according to the everyday clinical practices of dental clinics. The assessment was also performed as part of the practitioners’ routine activity.

A large proportion of published studies are constrained by numerous limitations, owing mainly to the replication of the standard design of traditional clinical trials, for example, the inclusion and exclusion criteria that are applied are usually very strict, as the condition of oral tissues must meet certain standardized specifications or must have very well-defined defects [28]. These constraints alone make it difficult to extrapolate the findings to the reality of daily dental practice or to the general population. Among the many biases that can occur, follow-up bias is often encountered: patients who participate in these studies are usually extremely controlled by the mere fact of having been selected. By contrast, the selection of patients in observational studies is not as restrictive and treatment is guided by the routine daily practice determined by the clinician or clinicians participating in the study.

In this study, the favorable results obtained should basically be attributed to the suitability of the Ocean implant system and the skills of the practitioners. It is possible that in other health care situations, with other less-skilled practitioners and/or with less clinical experience, the results would not have been the same [29] and survival would have been lower.

In the 24 months of follow-up after implant loading, 9 failures were recorded. The 1.6% rate of early failures—osseointegration period—is similar to that described in similar studies, both using controlled and uncontrolled [9,30]. That is, 6 implants were lost in the first six months and only three in the remaining 24 months.

The cumulative survival rate was 98.3%. The analysis of the variables that could be related to implant loss did not identify any that would in itself explain treatment failure. Neither the implant morphology nor the surgical technique or surface properties can explain the failed cases. We can therefore say that the results obtained are independent of the variables described above.

The majority (33%) of patients only required a single implant and only 7.1% had six or more implants, although the scientific literature indicates that the treatment with the most predictable improvement of oral health-related quality of life (OHRQoL) is implant-supported or retained complete dentures [31]. With regard to length and diameter, the preferred implants were 4.0 mm in diameter and 11.5 mm in length. Although the scientific literature validates their rate of survival [32], wider implants were only placed in 3.1% of cases.

Both histologically and experimentally, it has been shown that the surface of implants with a specific micro-topography design, as in the case of Biomimetic Ocean implants, can result in a higher bone to implant contact with a much faster healing period than that necessary with older machined implants [33]. The benefits of these surfaces must first be demonstrated in vitro and subsequently in vivo, in order to have scientific and clinical validity. This is what has been done with the Biomimetic Ocean implant by Avinent Implant System in this multicenter study. It was observed that the implants with Biomimetic Advanced Surface were clinically osseointegrated in accordance with presently required implant quality standards, which would be consistent with the high survival rate observed in this study.

Osseointegration dental implants are available in different materials, body shapes, surface properties and coatings. Numerous surface modifications including turned, blasted, acid-etched, porous-sintered, oxidized, plasma-spayed surfaces, etc., or a combination of these procedures have been developed and are currently used with the aim of enhancing clinical performance. It has been shown that a prerequisite for a successful implant is a rapid osseointegration and a long-term stability. In both cases, the implant surface is very important. A chemically-modified surface has beneficial effects and significant improvements in the cell response and various surface modifications have been described in the literature [26,34].

The Biomimetic Advanced Surface (BAS) treatment has been obtained by a combination of two processes. First, a shot blasting procedure was carried out using aluminum oxide as a blast media. Afterwards, the implants were anodized using an electrolyte solution rich in calcium and phosphate. The BAS surface topography is characterized by the presence of macroroughness and microporosity in the titanium oxide with a calcium and phosphorus deposit [26,34].

Although we are conscious that this study was the first evaluation of this type of implants, and that further studies with larger sample sizes and longer follow-up will be necessary, the results obtained in this study allow us to conclude that implants with a modified calcium-phosphate surface are associated with a high rate of survival and may be considered a method of choice in clinical practice.

## 4. Materials and Methods

### 4.1. Study Design

A multicenter, prospective, non-interventional, observational study was conducted to evaluate the survival of Biomimetic Ocean implants (Avinent Implant System, S.L., Barcelona, Spain), which had a patient inclusion period of three years (November 2011–November 2014) and a 24-month follow-up after implant loading.

### 4.2. Ethics Statement

The study procedures were performed following to the ethical principles of the Declaration of Helsinki and its subsequent revisions. All the subjects participating in the study gave their informed written consent to participate in the study prior to inclusion and patient data were obtained from the clinical records and entered in an archive with an anonymization procedure allowing patient anonymity to be maintained.

### 4.3. Patients, Clinicians, and Implants

The patients included belonged to three private Spanish dental clinics (Barcelona, Girona, and Madrid) and were over 18 years of age, with an indication for the insertion of one or more implants to replace missing teeth. The presence of clinical conditions that contraindicated any type of oral surgery was a criterion for exclusion.

After reading and signing the informed consent, all patients were assessed and treated for any oral diseases that they presented. Likewise, the presence of systemic risk factors (metabolic diseases, taking of prescription and nonprescription medication, alcohol and illicit drug use, radiation treatments, oncological surgery, chronic illnesses and physical disabilities, among others) was noted.

Three experts—two oral surgeons and one oral and maxillofacial surgeon—who were familiar with the implant system and certified in Spain to perform dental implant surgery and who have more than 15 years of experience participated in the study.

Implants with standard external or internal hexagon connections (Biomimetic Ocean by Avinent Implant System), having the same external geometry were used.

The implant bodies had diameters of 3.5, 4.0, 4.5, and 5.0 mm and lengths of 7.0, 8.5, 10.0, 11.5, 13.0, and up to 15.0 mm.

After the implants were integrated, a wide variety of prosthetic solutions by Avinent Implant System were used for the rehabilitation.

No specific protocols were recommended either for implant placement or loading. However, in the surgical phase, the position, type of implant to be placed and position of the platform were specified along with other data. Loading protocols were classified according to the criteria defined by Cochran et al. [35]: immediate restoration (placement within a maximum of 48 h of surgical implantation), early loading (placement of the restoration between 48 h and three months after implant surgery) and conventional loading (restoration is placed as a second step, after a healing period of three to six months after the implant surgery).

The implants were loaded with single crowns that were cemented or screw-retained, splinted to other implants as fixed partial or complete dentures or overdentures. All prosthetic components were used according to standard guidelines and the manufacturer’s instructions for use. Implant loading protocols were determined according to the criteria used at each of the participating clinics.

During the first stage of treatment planning, the results of the radiographic examinations and computerized tomography (CT, ProMax3DMid, Planmeca, Helsinki, Finland) scans (when performed), as well as oral and clinical characteristics of the patients were assessed and surgery was scheduled.

Surgery was performed in the second stage, after which the following was recorded: patients gender and age, implant position, dimensions, connection (internal hexagon (IH) or external hexagon (EH)), bone density according to the Lekholm and Zarb classification system [36] (types I, II–III, IV), characteristics of the bone (healed bone, post-extraction or regenerated bone), regeneration technique (performed before or during surgery), final position of the platform (supracrestal, crestal, or subcrestal), surgical technique (one-stage, when there was sufficient primary stability (>35 Ncm), or two-stage, in which case the implant was left submerged for at least six months), and loading type (immediate, early or delayed, based on the criteria described by Cochran et al.) [35].

Suture removal was performed 7–14 days after surgery. During the check-up visit prior to loading, each implant was evaluated for various clinical and radiological aspects that could lead to a suspicion of potential failure such as the possible presence of mobility, bleeding on probing, plaque index, and/or bone loss.

After implant placement, the design of the prosthesis was done using computer-aided design (CAD)-computer-aided manufactured (CAM) or conventional techniques; the type of restoration (single or multiple, cemented or screwed, or direct to implant overdenture), material and manufacturing process (milled titanium, milled, or cast cobalt-chromium, or other materials) were recorded.

A radiological evaluation after the implant placement and during follow-up (at one year and at two years) was made in order to measure bone levels.

Follow-up was conducted for 24 months after loading through clinical visits, periapical and panoramic radiographs with the corresponding analysis of implant function, during which the complications involved in the process were noted. The follow-up schedule was divided into six predefined periods: before surgery, after implant placement, after loading, six months later, one year later and two years later.

### 4.4. Study Objectives and Variables

Implants were considered to have survived if they were at their site of placement and functioned at follow-up control visits. Any implants that were lost spontaneously or extracted after placement were recorded as failures.

Failures were classified as early when they occurred during the healing period and before loading, or late, when they occurred after the prosthesis had already been attached to the implant.

Implants of patients who stopped coming to their follow-up visits were recorded as dropouts.

The primary objective of the study was to evaluate the effectiveness of the implant or its survival at 2 years, using the Kaplan-Meier method to calculate the interval survival rate (ISR) and the cumulative survival rate (CSR), with the implant as the unit of analysis. ISR represents the proportion of implants in a group that survive during a defined or specific time interval [37]. CSR represents the proportion of items existing at the beginning of a time interval that survive until the end of the interval being studied [38,39].

Bone density was assessed according to the Lekholm and Zarb classification system (types I, II–III, IV) [36].

According to this classification, in Type I, the entire bone is composed of very thick cortical bone; in Type II, thick layer of cortical bone surrounds a core of dense trabecular bone; in Type III, a thin layer of cortical bone surrounds a core of trabecular bone of good strength; and in Type IV, there is a very thin layer of cortical bone with low density trabecular bone of poor strength [40].

Assessment of implant stability, including assessment of regeneration, was evaluated using standard techniques [41].

### 4.5. Statistical Analysis

A descriptive analysis of measures of central tendency (mean, median) and dispersion (interquartile range and standard deviation) was performed for quantitative variables according to normality criteria (Kolmogorov-Smirnov test, *p* > 0.05), for both the participants in the study and the implants, and qualitative variables were described by frequency and percentage. The Kaplan-Meier test was used to assess implant survival and survival was compared over time with the log-rank test; 95% confidence intervals (95% CI) were presented. In every case, a *p*-value <0.05 was considered to be statistically significant. The Statistical Package for the Social Sciences (SPSS) version 17.0 for Windows (SPSS, IBM, Chicago, IL, USA) was used.

## 5. Conclusions

Implants with a modified calcium-phosphate surface are associated with a high rate of survival and may be considered a method of choice in clinical practice.

## Figures and Tables

**Table 1:** Characteristics of the study population (n = 212).

Patients’ Characteristics		n = 212	%
Age, mean (SD)		51.2	(11.9)
Gender, n (%)	Men	109	51.5
	Women	103	48.5
Implants per patient, median (IQR)		2.0	(2.0)
Smoking status, n (%)	Smoker ≥ 10 cigarettes/day	20	9.4
	Smoker < 10 cigarettes/day	20	9.4
	Never smoked	172	81.1
Periodontal disease, n (%)	No	148	69.8
	Yes; controlled	64	30.2
Bruxism, n (%)	No	136	64.2
	Yes	76	35.8
Number of implants per patient, n (%)	1	70	33.0
	2	67	31.6
	3	27	12.7
	4	21	9.9
	5	12	5.7
	6	8	3.8
	7	2	0.9
	8	1	0.5
	9	1	0.5
	12	3	1.4

SD: Standard deviation; IQR: Interquartile range.

**Table 2:** Types of connection, diameters and lengths (n = 544).

Characteristics of the Implants		n	%
Connection, n (%)	External hexagon	144	26.5
	Internal hexagon	400	73.5
Diameter, n (%)	3.5 mm	153	28.1
	4.0 mm	185	34.0
	4.5 mm	189	34.7
	5.0 mm	17	3.1
Length, n (%)	7.0 mm	1	0.2
	8.5 mm	17	3.1
	10.0 mm	155	28.5
	11.5 mm	233	42.8
	13.0 mm	138	25.4
Bone density, n (%)	Type I	44	8.1
	Type II–III	430	79.0
	Type IV	70	12.9
Bone characteristics, n (%)	Extraction site	36	6.6
	Healed bone	378	69.5
	Regenerated bone	130	23.9
Surgical stages, n (%)	1 stage (healing abutment)	89	16.4
	2 stages (cover screw)	455	83.6
Type of loading, n (%)	Immediate (<48 h)	32	5.9
	Early (8–12 weeks)	33	6.0
	Delayed (>12 weeks)	479	88.1
Final position of the platform, n (%)	Subcrestal	29	5.3
	Crestal	510	93.8
	Supracrestal	5	0.9

**Table 3:** Type of prosthetic restoration and material of the prosthesis (n = 504).

Prosthesis Characteristics		n	%
Material of the prosthesis, n (%)	Cast Cobalt-Chromium	212	42.1
	Milled Cobalt-Chromium	266	52.8
	Milled Titanium	6	1.1
	Others	20	4.0
Type of prosthetic restoration, n (%)	Screw-retained crown	146	29.0
	Screw-retained bridge	273	54.2
	Cement-retained crown	6	1.2
	Cement-retained bridge	24	4.8
	Overdentures	23	4.6
	Others	32	6.3
Design of the prosthesis, n (%)	CAD-CAM	266	52.8
	Conventional	238	47.2

**Table 4:** Characteristics of failures documented during the study.

Failure	Position	Months of Follow-Up	Implant	Bone Density	Bone Characteristics	Periodontal Disease	Bruxism	Smoking	Age
1	23	3	3.5 × 13 IH	Type II–III	Regenerated bone	Yes	No	Non smoker	76
2	31	13.70	3.5 × 13 IH	Type II–III	Regenerated bone	No	Yes	Non smoker	43
3	47	4.27	4.0 × 10 IH	Type II–III	Healed bone	No	No	Non smoker	62
4	31	5	3.5 × 11.5 IH	Type II–III	Alveolar after extraction	No	Yes	Non smoker	45
5	26	0.23	4.0 × 10 IH	Type II–III	Healed bone	No	No	Non smoker	60
6	26	3.30	4.5 × 10 IH	Type II–III	Regenerated bone	Yes	No	Non smoker	64
7	36	18.63	4.5 × 10 IH	Type II–III	Regenerated bone	Yes	No	Non smoker	57
8	46	6.53	4.0 × 10 IH	Type II–III	Healed bone	No	Yes	Non smoker	35
9	37	4.20	4.0 × 11.5 IH	Type II–III	Healed bone	Yes	Yes	Non smoker	44

**Table 5:** Cumulative survival from start to end of follow-up (n = 544) and interval survival.

	No. of Implants at Risk	No. of Failures	No. of Lost Implants	Cumulative Survival	Lower Limit 95% CI	Upper Limit 95% CI	Interval Survival	Lower Limit 95% CI	Upper Limit 95% CI
**0–3 months**	544	2	5	99.63	99.12	100.00	99.63	99.12	100.00
**3–6 months**	537	4	64	99.04	98.22	99.87	98.89	98.00	99.78
**6–9 months**	469	0	7	99.04	98.16	99.93	100.00	--	--
**9–12 months**	462	0	12	99.04	98.16	99.93	100.00	--	--
**12–15 months**	450	1	6	98.82	97.82	99.82	98.67	97.61	99.73
**15–18 months**	443	1	53	98.57	97.47	99.67	98.44	97.29	99.59
**18–21 months**	389	1	54	98.32	97.04	99.60	98.18	96.85	99.51
**21–24 months**	334	0	30	98.32	96.94	99.70	100.00	--	--

**Table 6:** Survival from start of follow-up to end of follow-up in terms of various risk factors (n = 544).

Patient Characteristics		Cumulative Survival at 24 Months (95% CI)	Log-Rank *p*-Value
Age	<65 years	98.14 (96.42–99.86)	0.413
	≥65 years	99.02 (96.68–100.00)	
Smoking Status	Never smoked	97.86 (96.02–99.70)	0.226
	Smoker < 10 cigarettes/day	100.00 (-)	
	Smoker ≥ 10 cigarettes/day	100.00 (-)	
Periodontal disease	No	98.49 (96.84–100.00)	0.339
	Yes	97.93 (95.04–100.00)	
Bruxism	No	98.57 (96.99–100.00)	0.246
	Yes, with/without bite splint	97.73 (94.60–100.00)	
Position of the implant	Mandible	97.35 (94.89–99.81)	0.136
	Maxilla	99.06 (97.63–100.00)	
Implant connection	External hexagon	99.26 (96.86–100.00)	0.785
	Internal hexagon	98.11 (96.44–99.78)	
Bone density	Type I	97.14 (90.73–100.00)	0.911
	Type II-III	98.16 (96.45–99.87)	
	Type IV	100.00 (-)	
Bone characteristics	Extraction site	96.67 (88.81–100.00)	0.160
	Healed bone	99.11 (97.87–100.00)	
	Regenerated bone	96.29 (91.70–100.00)	
Surgical stages	One stage (healing abutment)	95.72 (89.60–100.00)	0.268
	Two stages (cover screw)	98.78 (97.45–100.00)	
Type of loading	Immediate (<48 h)	100.00 (-)	0.255
	Early (8–12 weeks)	96.77 (93.34–100.00)	
	Delayed (>12 weeks)	98.37 (96.82–99.92)	
Final position of the platform	Subcrestal	100.00 (-)	0.699
	Crestal	98.20 (96.66–99.74)	
	Supracrestal	100.00 (-)	
Diameter of the implant (mm)	3.5	97.90 (95.45–100.00)	0.869
	4.0	98.27 (96.22–100.00)	
	4.5	98.51 (96.19–100.00)	
	5.0	100.00 (-)	
Length of the implant (mm)	7.0	100.00 (-)	0.322
	8.5	100.00 (-)	
	10.0	97.97 (95.60–100.00)	
	11.5	99.54 (98.64–100.00)	
	13.0	99.26 (97.81–100.00)	
Number of implants	1	93.26 (86.31–100.00)	0.157
	>1	97.06 (93.92–100.00)	
Number of implants	1	93.26 (86.31–100.00)	0.205
	2–6	97.60 (94.01–100.00)	
	>6	87.50 (63.00–100.00)	
